# Public-private knowledge transfer and access to medicines: a systematic review and qualitative study of perceptions and roles of scientists involved in HPV vaccine research

**DOI:** 10.1186/s12992-020-00552-9

**Published:** 2020-03-05

**Authors:** Rosa Jahn, Olaf Müller, Stefan Nöst, Kayvan Bozorgmehr

**Affiliations:** 1 Department of General Practice and Health Services Research, University Hospital Heidelberg, Im Neuenheimer Feld 130.3, 69120 Heidelberg, Germany; 2 Heidelberg Institute of Global Health, University Hospital Heidelberg, Im Neuenheimer Feld 130.3, 69120 Heidelberg, Germany; 3 Department of Population Medicine and Health Services Research, School of Public Health, Bielefeld University, P.o. Box 10 01 31, D- 33501 Bielefeld, Germany

**Keywords:** Public-private technology transfer, University innovation, Access to medicines; medical research and development, Prophylactic HPV vaccines

## Abstract

**Background:**

Public research organizations and their interactions with industry partners play a crucial role for public health and access to medicines. The development and commercialization of the Human Papillomavirus (HPV) vaccines illustrate how licensing practices of public research organizations can contribute to high prices of the resulting product and affect accessibility to vulnerable populations. Efforts by the international community to improve access to medicines have recognised this issue and promote the public health-sensitive management of research conducted by public research organizations. This paper explores: how medical knowledge is exchanged between public and private actors; what role inventor scientists play in this process; and how they view the implementation of public health-sensitive knowledge exchange strategies.

**Methods:**

We conducted a systematic qualitative literature review on medical knowledge exchange and qualitative interviews with a purposive sample of public sector scientists working on HPV vaccines. We explored the strategies by which knowledge is exchanged across institutional boundaries, how these strategies are negotiated, and the views of scientists regarding public health-sensitive knowledge exchange.

**Results:**

We included 13 studies in the systematic review and conducted seven semi-structured interviews with high-ranking scientists. The main avenues of public-private medical knowledge exchange were publications, formal transfer of patented knowledge, problem-specific exchanges such as service agreements, informal exchanges and collaborative research. Scientists played a crucial role in these processes but appeared to be sceptical of public health-sensitive knowledge exchange strategies, as these were believed to deter corporate interest in the development of new medicines and thus risk the translation of the scientists’ research.

**Conclusion:**

Medical scientists at public research institutions play a key role in the exchange of knowledge they generate and are concerned about the accessibility of medicines resulting from their research. Their scepticism towards implementing public health-sensitive knowledge management strategies appears to be based on a biased understanding of the costs and risks involved in drug development and a perceived lack of alternatives to private engagement. Scientists could be encouraged to exchange knowledge in a public health-sensitive manner through not-for-profit drug development mechanisms, education on industry engagement, and stronger institutional and legal backing.

## Background

Public research organizations (PROs) play a crucial role in the advancement of medical science [1]. Of all scientifically novel medicines approved by the U.S. Food and Drug Administration (FDA) between 1998 and 2007, one in five were developed by PROs [1]. Medicines submitted by PROs were also more likely to receive priority review, awarded by the FDA for medicines deemed to represent major advances of the medical practice. Almost half of all drug approvals submitted by PROs have received priority review, compared to one in five submissions from the private sector – indicating that research carried out at PROs has a higher public health impact [2].

The contribution of PROs is particularly significant in the field of vaccines – virtually all important and innovative vaccines approved from 1985 to 2010 have been developed by PROs [2]. These include the vaccines against Human Papillomavirus (HPV). HPV infection is the main cause of cervical cancer [3, 4], which ranks fourth for cancer incidence and mortality among women, with 570,000 cases and 311,000 deaths worldwide in 2018 [5]. It is the leading cause of cancer death in women in mainly sub-Saharan Africa and South-Eastern Asia, thus disproportionately affecting young women in regions where screening and treatment options are limited [4–7].

In the 1980s, Prof. Harald zur Hausen, then working at the publicly-funded German Cancer Research Center (DKFZ), discovered the association between HPV infection and cervical carcinoma [8]. The first vaccine candidates were based on further research conducted by groups at the University of Queensland, Rochester University, the U.S. National Cancer Institute (NCI) and Georgetown University with contributions from other PROs such as the DKFZ [9–15]. These research groups then licensed their Intellectual Property (IP) to MedImmune, GlaxoSmithKline (GSK) and Merck, Sharpe & Dohme (MSD) for further development and clinical testing for efficacy and safety. Except for the licenses acquired from the NCI, these were all exclusive licenses, allowing the licensee to exclude others from making, marketing or selling the patented material MedImmune and GSK later formed a vaccines alliance and shared their IP with each other. In 1997, the U.S. Patent and Trademark Office initiated patent interference proceedings between research groups in Queensland, Rochester, the NCI and Georgetown. As part of the dispute settlement the non-exclusive licenses provided by the NIH were turned into co-exclusive licenses in 2007. Both MSD and GSK had already cross-licensed their IP to each other, guaranteeing themselves unhindered access to the HPV vaccine technology and securing their market positions [16–18]. In 2006 and 2007, the European Medicines Agency approved two prophylactic HPV vaccines: Gardasil® (MSD) and Cervarix® (GSK).

The HPV vaccines were later included in the World Health Organization’s Essential Medicines List, which outlines the “minimum medicine needs” of populations [19]. Recognizing the human right to the enjoyment of the highest attainable standard of health, the international community has committed to providing “access to affordable essential medicines and vaccines for all” [20–23]. However, in 2014, only 1.2% of females 10–20 years of age in Africa and 1,1% of the same population in Asia had been fully immunized, despite accounting for 70% of all HPV-related cancer cases [24–26]. As of June 2018, only seven African and two South-East Asian countries have implemented national immunization programs for HPV [27], indicating that immunization coverage fails to meet global needs even 12 years after the first vaccines became available. The reluctance of country governments to include HPV in national immunization programs has been associated with the high price of the vaccines, especially for states in transition that do not qualify for support mechanisms such as GAVI [26, 28–30]. However, the production costs for HPV vaccines are estimated to be as low as 3 USD for affluent and 0.60 USD per dose for less affluent markets respectively [31], and by 2014, HPV vaccine sales had already amounted to a total of USD 14.1 billion, recouping the investment made by the pharmaceutical companies fivefold [32]. Prolonging the status quo, recent patent applications filed by GSK are likely to delay market entrance of cheaper generic products until 2020 [31, 33].

As illustrated above, scientists and their public institutions play a crucial role not only in the development of medicines and vaccines but their knowledge exchange practices with private companies also shape the availability and accessibility of the resulting drugs. The Sustainable Development Goals, the WHO’s draft road map for access to medicines, vaccines and other health products as well as Reports by the UN High-level Panel on Access to Medicines and the Lancet Commission on Essential Medicines have emphasized the need to rethink the management of publicly funded innovation in a manner that “promotes access to health products” [21–23, 34]. The implementation of public health-sensitive knowledge management has also been supported by NGOs, such as Universities Allied for Essential Medicines (UAEM) [16].

The strategies put forward by the reports and organizations named above aim to promote access to medicines mainly through the public health-sensitive transfer of patented technologies [21, 35, 36], such as non-exclusive licenses or obligations regarding affordability and accessibility of the final product [37–40]. While such formal transfer of intellectual property constitutes a relevant and easily targeted knowledge exchange mechanism, it is far from the only one [41, 42]. Bradley, Hayter and Link formulate a broader concept of technology transfer, which includes the traditional licensing pathway through the PRO’s Technology Transfer Office (TTO) as well as informal transfer mechanisms such as collaborations and informal exchange of knowledge [43]. Exploring these less codified forms of communication and emphasizing the role of the originator scientist, Perkman et al. have proposed the concept of academic engagement, any “knowledge related collaboration” by scientists at PROs with private entities [44]. Both technology transfer and academic engagement are set within the broader framework of knowledge generation and diffusion, comprehensively and famously conceptualized in the Triple Helix. In this model, knowledge is exchanged across institutional boundaries through an overlay of formal and informal, explicit and implicit communications between the private, academic and government spheres [41–45]. To encompass all avenues of communication, the term ‘knowledge exchange’ will be adopted for this paper.

However, in contrast to the growing political interest in knowledge exchange practices of PROs in the context of access to medicines, as well as substantive theoretical literature; empirical research on public-private knowledge exchange in medical research remains scarce. To our knowledge, neither a review of the current literature nor an exploration of the perspectives of scientists at PROs regarding the possibility of pursuing public health-sensitive knowledge exchange, has been conducted so far. Such knowledge would serve to better support the efforts within the global access to medicines community to identify and implement knowledge exchange strategies that promote access to medicines.

The objectives of this paper are to explore (1) how medical knowledge is exchanged between PROs, particularly individual inventor scientists’, and private actors, and (2) which public health-sensitive knowledge exchange strategies inventor scientists are aware of and how they view their implementation.

## Methods

To address the objectives of this paper, we conducted a systematic review and a qualitative study on the development of the HPV vaccines.

### Systematic review

Before conducting the systematic review, we published a study protocol outlining our search and analysis strategy [46]. The review addressed how medical knowledge is exchanged between PROs and private actors, and in particular how scientists engage in the process. The review included the databases PubMed and Web of Science as well as ProQuest, DiVa and DART Europe. The search terms covered medical public research and technology transfer or research collaborations. The search terms were developed based on the PICo approach, whose components are population (P), phenomenon of interest (I) and context (Co) [47]. The initial search terms were tested and adapted to each database [46].

Included were studies addressing any kind of formal or informal exchange of knowledge from the public to the private sector and focusing on medical research. We included studies published since 1995 to capture the period after the Agreement on Trade-Related Aspects of Intellectual Property Rights (TRIPS) entered into force. The literature under review was limited to qualitative studies as this type of research “is most revealing when the variables of greatest concern are unclear” [48]. Excluded were commentaries; theoretical texts; books and meeting reports as well as studies focusing on an industry perspective or knowledge exchange effectiveness.

The screening strategy was piloted by screening one-fifth of the studies in duplicate (RJ, KB). All disagreements between the screening results were discussed by RJ, KB, and SN. The remaining studies were screened by RJ in close consultation with the research team. All included studies were assessed for their quality using the qualitative research checklist developed by the Clinical Appraisal Skills Programme (CASP) [49]. Data extraction and evidence synthesis followed the meta-ethnography approach first developed by Noblit and Hare [50]. It describes the identification and comparison of major themes, “metaphors”, across studies and is particularly suitable when a wide variety of studies is expected. The study findings were identified using open coding in MAXQDA. After coding the first two studies and discussing the results in the research team, the extraction strategy was adjusted to better capture all relevant findings. Coding and analysis of the remaining studies were then performed by the first author. The codes and synthesis based thereon were discussed and refined within the research team. Further details of all steps of the systematic review are provided in the protocol [46].

### Qualitative HPV case study

Between March 2014 and March 2015, we conducted seven qualitative, semi-structured in-depth interviews with a purposive sample of high ranking scientists from four countries working on prophylactic HPV vaccines. The interviews addressed the knowledge exchange between public and private entities in HPV research in general as well as opportunities regarding the promotion of access to medicines. HPV was chosen as a case study because, as described above, it provides insights into the exchange of knowledge at the intersection of private and public interests which is at the heart of the access to medicines debate.

**Fig 1:**
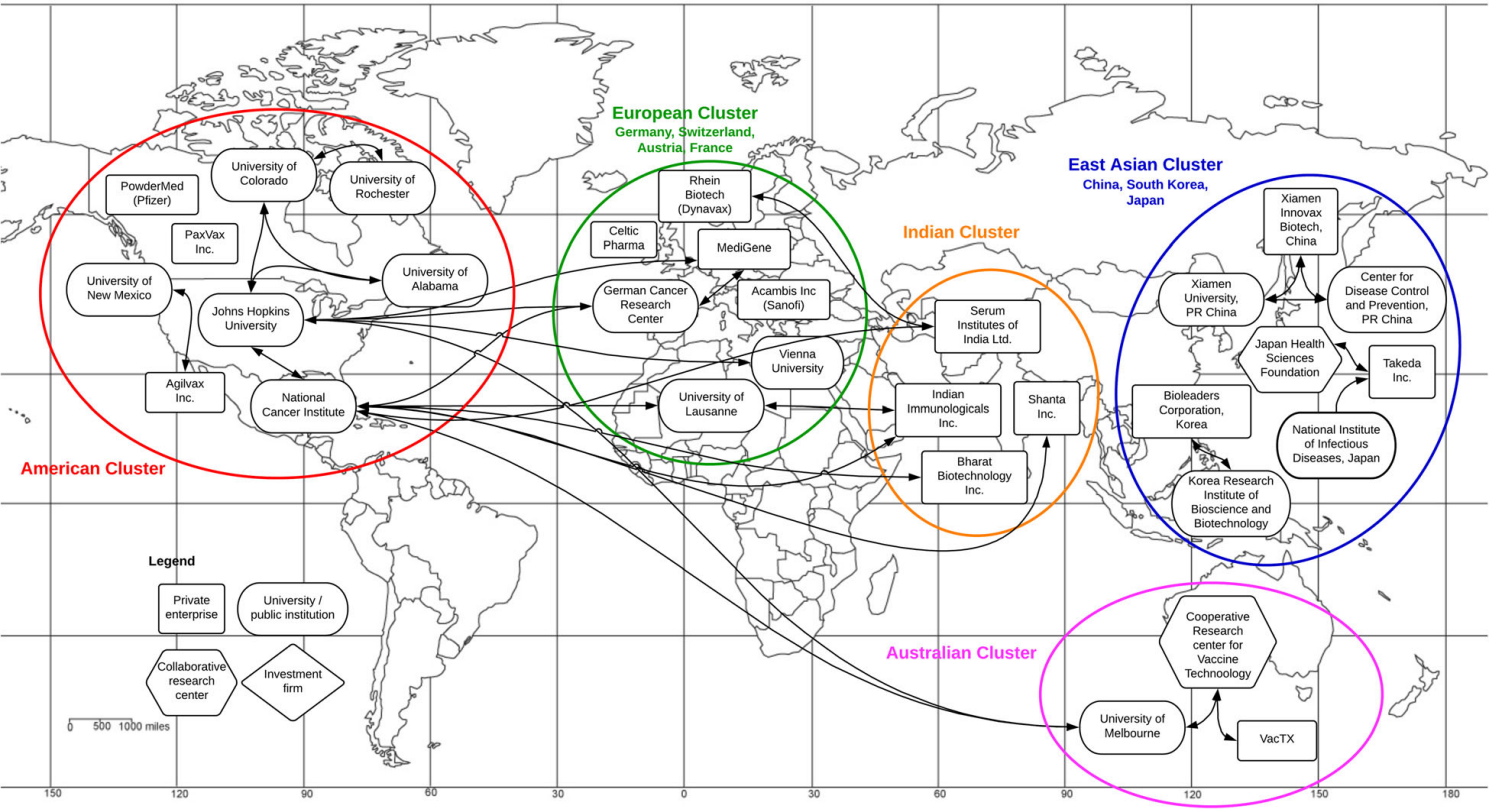
Actors in HPV vaccine research, by geographic location (2014)

In the initial phase of this study, we conducted a review of scientific publications on HPV vaccines as well as university and government websites to identify institutions, researchers and collaborations currently engaging in prophylactic vaccine research (see Fig. 1) [17, 51–62]. This research showed regional clusters of institutions currently exploring new or improved versions of prophylactic HPV vaccines. The clusters in the U.S and Europe as well as, to a lesser extent, those in the U.S. and Australia, were found to be engaging in joint research, whereas the East Asian cluster appeared to be somewhat isolated. The institutions in India we identified through our literature search were exclusively private companies, which were engaging with PROs from the U.S. and European clusters. We used a purposive sampling strategy, aiming for diversity with regards to the geographical region, kind of research collaboration, as well as gender and age of the scientist. We contacted 12 scientists working at three PROs from the European, four from the American, one from the Australian, and two from the East Asian cluster. Of the 12 scientists, seven agreed to participate.

The interviews were conducted using an interview guide, developed by the research team based on the health policy triangle described by Gilson and Walt [63]. It addressed the actors involved in knowledge exchange and their power, the negotiation process, as well as exchange strategies and the broader context. The views regarding access to medicines were addressed within these categories. The interview guide was piloted in a test interview and adapted during the course of the interviews to reflect perspectives gained and emerging theory (see Appendix A for the first and final version).

Two interviews were conducted in the workplace face-to-face and five via Skype. With the interviewee’s consent, all interviews were recorded. Important points were noted during the interview and a post scriptum was written shortly afterwards. The interviews lasted between 45 and 90 min. All interviews were conducted, transcribed and coded in MAXQDA by RJ in English and German (for coding tree see appendix B).

The analysis of the interview data was based on the method of framework analysis [64] because it allows for the generation of new theories from the data while incorporating previous knowledge into the design and analysis of the study. Every participant was assigned a pseudonym by randomly choosing one of the 35 most common surnames in the United States while maintaining the respective academic grades: Dr. Williams, Prof. Thomas, Dr. Jones, Prof. Smith, Prof. Taylor, Dr. Wright and Dr. Wilson. Our literature review revealed that very few women are active in HPV vaccine research. To ensure that no female participants can be identified, we thus only use the male form in the presentation of our results. Likewise, we limit the description of the study population, including place of work, details of research projects or geographic area to avoid identification of individual participants.

## Results

### Systematic review

The search strategy yielded 1380 results. After removing all duplicates, 1257 studies remained and were screened based on abstract and title. 112 studies were included for full-text review, six of these could not be retrieved and 93 were excluded (see appendix C for PRISMA flow chart). 13 studies were included in our analysis, 11 journal papers and two dissertations [65–77]. The quality of included studies ranged from five to nine points, indicating moderate to high quality (for a detailed description see annex C).

### Knowledge exchange strategies and negotiation

The reviewed studies described various methods of exchanging knowledge, including academic publications and the presentation of research findings at conferences. Often in contrast to these “academic channels” [66], studies discussed the protection of IP through patents and the subsequent licensing of the protected technologies [65, 67, 74]. Two papers, in particular, explored the conflict between publishing and patenting knowledge [66, 67]. They concluded that peer-reviewed publications function as a signal to the academic community that research is scientifically valuable. Individual scientists, their institutions, as well as early-stage spin-off companies, tend to signal their academic legitimacy through publications. Patents, on the other hand, signal commercial value and are thus preferred by pharmaceutical and biotech companies as well as spin-offs that are already commercially exploiting their technology. A more recent study, however, found that patents and commercial success also translate into academic prestige, indicating a shift towards a more positive view of industrial engagement among the academic community [68]. It was also reported that some PROs provide financial incentives to scientists engaging in patenting and commercialization [68]. The only study from the African continent described that in Tanzania, public sector scientists avoided filing patents because the patent enforcement mechanisms were perceived to be too weak to adequately protect the IP disclosed in the patent application [76].

The only type of license discussed in the reviewed studies was the exclusive license. Ard (2002) found that scientists believed engaging with a company to be necessary for developing their research into products, and that exclusive licenses were the only way to encourage industry involvement [65].

Spin-off companies were discussed in-depth by Bacevice (2010), who suggests that these can take different organizational forms. Number and geographical proximity of members, the involvement of other companies and funders, connections between the spin-off, the founding scientist and the originator institution as well as communication formats vary greatly [66]. The sustained involvement of the inventor scientists may be influenced or curtailed by funders, venture capital firms, for example, were reported to often request a change of leadership [75]. Academic scientists were found to also be involved in consulting activities or contract research that lead to a formalized knowledge transfer between academia and industry. These include clinical studies [69], provision of equipment, in-kind counselling activities, assistance in problem-solving or investigation of novel problems [71].

By far the most extensively explored forms of knowledge exchange were collaborative research endeavours. Two studies independently identified four phases of network development [70, 71], with other studies providing additional detail (see Table 1). These studies show that the knowledge exchange strategies used by networks vary with the network development stage. They often begin with informal, personal contacts initiated by scientists working at PROs, for example at conferences. Once a rapport is established, a formal framework is negotiated between the partners and with the involvement of the PRO’s TTO. The further the network progresses, the more trust is established and contractual routines are relaxed. Informal transfer of implicit knowledge between the partners then becomes possible. During the more advanced stages of network development, patentable IP may be produced and transferred formally. Especially in the initiation of research networks, scientists were found to play a key role.

**Table 1:** Four Phases of Network Development

Phase I	A few individual key actors initiate or renew connections among scientists working on related issues [70, 71]. These actors are often academics or government scientists who reach out to industry with an idea for a research project [68, 72]. In this phase, knowledge exchange occurs through publishing and presenting papers, conferences and a few proprietary projects between individual scientists and individual firms [71].
Phase II	After the key actors receive a positive response from their partners, a formal agreement must be reached [70], including an IP strategy [71, 73] and clearly identified objectives. Subsequently, partners need to commit to the network by providing or obtaining funding, which is then invested to stabilize the network [70, 71]. Lastly, members of the new network develop a leadership structure, for example through a manager [71] or a board [70].
Phase III	This phase establishes routines of interaction, trust and knowledge sharing within the network [70, 71]. With increasing trust between the members of the network and faith in its success, contractual routines are relaxed and members become more agile in their interactions [71].
Phase IV	Phase four is the knowledge-generating phase [70]. The members of the network are exchanging explicit as well as tacit knowledge and contribute to new discoveries and their interpretation [71]. This success leads to further commitment by the members of the network and often sub-projects evolve around specific issues [70, 71].

### The inventor scientists’ view on public-private knowledge exchange

The scientists engaging in knowledge exchange with companies were described to often be “unusually successful, well placed, and highly visible” white men with PhD or MD degrees at major research institutions [67]. It emerged that while some scientists are wary of commercial engagement and are not taking active steps towards patenting or commercialization, others feel more comfortable crossing institutional boundaries and regularly engage with private partners [67]. These typologies were most extensively explored by Owen-Smith and Powell (2001), who developed four faculty types: the Old School Professor, the Reluctant Entrepreneur, the New School Professor and the Engaged Traditionalist. Realizing the societal benefit of their research, however, emerged as one relevant reason to engage in knowledge exchange with companies to develop marketable products. The motivating factors as well as perceived limitations of engaging with industry are summarized in Table 2.

**Table 2:** Motivating factors and perceived limitations in engaging with industry

Motivation	
Acquiring resources	“Human capital, equipment, and access to proprietary information”, funds for “post-docs” and “space” “feed back into better science” [67]
	Aquiring funds for “newer equipment for their laboratories” [68]
	Funds for their research” and “PhD projects and payments for lab tests, research materials, salaries, or conference travels.” [69]
Personal reward	Obtain a personal financial reward [68]
	Interviewees stress that they do not “get a penny” [69]
Societal Impact	“All of us know someone who has died of cancer. […] Fluorescent in situ hybridization is a way […] to help diagnose cancer. [...] I am very, very proud of what we have accomplished” [65]
	New School Professor: “I’ve had cancer twice. I’ve had many friends die from HIV. [...] (M)y research [...] deals with HIV and cancer. If I feel that I have an opportunity here to make a difference”, “collaborations between universities and industry will have positive societal benefits as they speed the discovery and development of new therapeutics” [67]
Others	Interesting work [69], Reputation with industry and learning opportunities [68], High-level influence in the respective field [66]
**Limitations**	
Conflict of Interest	“Virtually all [of 27 interviewees currently engaged in public-private partnerships] expressed concerns about conflict of interest related to private industry” [77]
	“I don’t care what they write into the contract in terms of independent reporting... the reality is that they’re not going to bite the hand that feeds them. [...]. It may not be intentional but the university will err on the side of being favorable to industry. We all know how you can paint a different picture with the same set of circumstances without ever lying.” [65]
Academic Freedom	Old school type: “There’s a certain greedy, ‘have it now’ mentality that may motivate people to try to get out there and do something dramatic from which they’re going to profit in a short time. Some people even choose their scholarly area in order to position themselves in that respect” [67]
	“If someone else files a patent that conflicts with your work, that could really impair your research” [67]
Research shift	Old School Type: “in biomedical science, there is a very widespread feeling that the higher quality you are, the more you’re going to be raking in, the more patents you’ll have, and the more companies you’ll be associated with. [...] There is a big reorganization under way such that traditional fields, small low funded fields that endow the institution with great diversity, are going by the wayside. What you’re going to wind up with are big juggernauts of work in a few areas like functional genomics.” [67]
Secrecy	Old School Type: “It’s anathema to me that you can find people in academic settings who won’t talk about what they’re doing. They can’t tell you what they’ve found because of patents, pending patents, or applications. If you can’t talk openly, it’s bad” [67]
	Reluctant Entrepreneur: “My lab generates knowledge that could be of great value to companies. Since it is not done in a company, the knowledge could be viewed as a loss to some firms. But we want to be able to publish it. So the company might have an incentive to restrict or control our research” [67]

### Knowledge exchange and access to medicines

Views on access to medicines or strategies to promote affordability of resulting products were not discussed by any of the reviewed studies, although patient benefit was discussed as a motivator for research and knowledge exchange. One study indicated that exclusive licenses are perceived to be the only viable licensing framework, as they provide companies with the necessary incentive to engage in the development of the patented technology [65].

#### Qualitative interview results

For the qualitative study of HPV vaccine development, we interviewed seven scientists, most of them high ranking, male and heading their own research units. Three interviewees worked at government institutions, four at public universities in Europe, America and Australia. The combined years of work experience of all participants was 188 years, the mean of individual HPV research experience was 27 years. All had filed at least four patents and licensed at least once. All non-European participants reported that they had started at least one spin-off company. All but one participant had been involved in the development of Gardasil® and Cervarix®; of these, most were still working on HPV vaccines at the time of this study.

### Knowledge exchange strategies and negotiation

The knowledge exchange strategies addressed by study participants included publication in journals or presentations at conferences; patents and subsequent licenses, sales or spin-off companies; service or research agreements; requests for advice; informal and personal exchange of knowledge as well as research collaborations.

Publications in the form of journal papers or conference contributions emerged as the most important currency in medical research. However, the interviews suggested that patents have become more common and are increasingly considered to be relevant within the scientific community, too. The scientists reported scanning knowledge for patentability before publication but said that determining whether a research finding may be patentable was often challenging.

“As a researcher, I don’t live off patents, I live off publications. [...]If you apply here somewhere, it’s primarily about peer reviewed articles.” (Prof. Smith)

“It’s become fashionable to patent. It seems like your research is more valuable if you have patents, and some people you know, want to patent, in part probably because they think they should because people are telling them to do it.” (Dr. Williams)

“It’s now considered a positive mark if you do do technology transfer and sort of a black mark if you [...] publish something when it could’ve been a valuable IP.” (Dr. Williams)

“In the beginning you may not even think that this is something patentable and you keep working on it and then [...] you are overcome be the realization that maybe you already said too much.” (Prof. Taylor)

Overall, the interviewed scientists described that they are encouraged by their PROs to explore patenting opportunities, but that the decision to disclose an invention to the TTO generally rests with the principal investigator, with a varying degree of input from the research team. All participants reported that, although it is not legally mandated to, the TTO usually followed through on patenting the disclosed invention and engaged the inventor scientists for ideas and suggestions regarding possible commercialization plans or potential commercialization partners. They are also usually the ones to establish contact with and present the research to interested parties. This was attributed mainly to the scientists’ experience and reputation within the HPV research field.

“Usually it’s driven by the investigator. If the investigator really thinks that this is going to have some kind of applicability, the technology transfer office will probably agree”. (Dr. Jones)

“You have to indicate in your invention report already, how could this potentially be marketed, who would be the potential partners who could market it“ (Prof. Taylor)

“What leads to success in the majority of cases, if at all, is that we refer to such companies. Why? Because the companies rather know [Prof. Smith] and [Prof. Taylor] and know what they do“. (Prof. Smith)

“We had meetings with people. So as the technology transfer arm of the university was trying to market the intellectual property [...] we had a lot of meetings. In some cases we would travel to companies, in some cases they would come here and then a lot of phone calls.” (Dr. Wilson)

The licenses that were negotiated were reported to be usually exclusive licenses. They were generally considered to be the only possible way to provide enough of an incentive for companies to engage in the commercialization of a technology. Some participants described specific licensing clauses, such as milestones, or non-exclusive licenses. While these were reported to encourage the working of the patent or access to research tools, none of the participants described licenses that promote access to medicines and did not think such licenses were feasible.

“And the vaccine, well, if we didn’t patent the vaccine to give some sort of exclusivity, noone can spend five hundred billion dollars developing it. Okay? There will never be the vaccine. So (that’s huge) for human health, even though there is some exclusivity, you have to do it or it just wouldn’t happen.“ (Dr. Williams)

“They say the state is financing the NIH, therefore the technology should be available to the citizens. [...] Patents are usually transferred non-exclusively, [...] so no monopolies are created.” (Prof. Taylor)

“One lever is for the university to include milestones. To say [...] if you don’t develop this within the next five years, you either have to pay us something, so a penalty in case [...] there is no development [...] or it falls back to us.“ (Prof. Taylor)

Negotiating licenses with firms was described to be hindered by TTOs, which were in some cases perceived to be understaffed and weak. In particular at public universities, which have tighter budgets than their private counterparts. The companies’ legal presence, on the other hand, was described to be much stronger.

“[P]rivate universities have more money to devote to their TTOs. [...] And plus the private universities can pay their TTO people more than the state university people so therefore they get better people. You know, so it’s, what can you say.” (Dr. Jones)

“[A] big company probably is more difficult […] to deal with because […] they have lawyers […]. The more lawyers there are involved the more it is difficult, that’s clear” (Dr. Wright)*.*

Instead of selling or licensing a patent, institutions and inventor scientists can decide to start their own spin-off company. All American interview participants mentioned that they had started at least one spin-off while none of the European participants had. One American participant stated that establishing a spin-off has become very common. Generally, scientists reported that a spin-off would be created to continue the development as long as possible until a favorable license or sale could be negotiated with a larger company. In this endeavor, they face limitations in how far they can take their technology because of financial constraints and participants generally believed phase II clinical trials to be too expensive for a spin-off.

“The other way of course is to start your own company, which a lot of academics are doing now and hopefully therefore can draw on private investors and things to develop their technology. So, I think that that at least in the United States, it has almost become epidemic” (Dr. Jones)*.*

„[T]he [spin-off] company is a small company and the way that these small biotech companies typically work is that smaller companies can often move products into phase one trials and sometimes into phase two trials, but at that point usually if the data looks promising the products get licensed to larger pharmaceutical companies“ (Dr. Wilson)

Other exchange pathways discussed by the participants included advice and service agreements as well as more collaborative research endeavors. Especially the participants who had been directly involved in the development of the HPV vaccine described that they are frequently being asked for advice. In this context, companies seem to be more interested in feedback on their work from experts in the field than acquiring new knowledge or technologies. Sometimes this exchange was facilitated through paid positions at the company, e.g. on advisory boards. Lastly, formal service agreements facilitate the exchange of knowledge or research materials between private companies and PROs. Similarly to the advice described above, companies often approach the scientists directly.

“They [...] commissioned quite a lot of work in that area from different groups, […] I mean they have their own expertise in-house obviously, but they wanted to be reassured by people who were outside of the company that they were getting it right.” (Prof. Thomas)

“And I work with several other companies on their scientific advisory boards or as [...] a board member to try and help them get commercialization and development, translation of the products out there into the real world.“ (Prof. Thomas)

“I’ve established a model […] of cancer […] So one company for instance contacted me because they wanted to learn how to make this model. So for this we had a service agreement and they came […] just two, three days just to learn how to make this particular model.” (Dr. Wright)

The interviews also shed some light on more collaborative forms of knowledge exchange. According to the interviews, research collaborations often come about through informal, unorganized interactions and knowledge exchange, connecting researchers from the public and private sector that are working on similar questions. This is facilitated by confidentiality agreements which are often signed at some point during the process and allow for free sharing of knowledge. While the subsequent formalization of a research collaboration is the mandate of the TTO, scientists described essentially developing preliminary terms with the partner and dictating these to the TTO.

“I believe most cooperations […] happen on a rather personal basis, so that you are in touch with two, three others you know personally and then you develop mutual trust and develop things together. And maybe commercialization is not in the forefront but rather how can we get this technology one step further?“ (Prof. Taylor)

“I talk to somebody from the company first and then if it looks like it’s going to be an interesting collaboration [...] I bring the tech transfer company in to help with that. [...]I might sort of set up a […] draft term sheet and say this is how I think they ought to do business and then they will go and sort of sort out the details and see if they can make it work.” (Prof. Thomas)

### The inventor scientists’ view on public-private knowledge exchange

The interview results suggest that the individual inventor scientists are indeed key actors in the exchange of the knowledge they create. The scientists also went into some detail regarding their views on knowledge exchange with private companies. They highlighted that involvement really depends on individual interest and commitment.

“I have kind of acquired this [interest in commercialization] epigenetically, others don’t have that as much” (Prof. Smith)

“It’s just who we are. I mean, [... ] we like to be in charge of our own developments. We were very interested in this whole process [...]. And we thought it was potentially important, so we didn’t want to lose control of it. It’s kind of the way we do things” (Dr. Williams)

An important motivator to engage in knowledge exchange and in commercialization in particular was to improve human health in general, promote public health in resource-poor settings or even to achieve universal access to the resulting product.

“I don’t see the point in developing things to cure mice; the point is to cure people.” (Dr. Wright)

„I was really keen to see the product used. Cervical cancer is […] really a disease of the developing world” (Prof. Thomas)

For the interviewed scientists, the impact on human health they were working for was mediated by developing products and bringing them to market. They all stated that to do so, the engagement with industry was a requirement, not a choice, because PROs were seen to lack the necessary technical and financial means. The drug development process was understood to be unbelievably expensive and thus far out of reach for PROs.

„Without a commercial partner there would be no vaccine. [...] if this was going to be […] useful then we needed somebody who was going to make it and sell it." (Prof. Thomas)

“Well, I mean obviously the ultimate goal is to improve human health. […] I feel like the company is really the fastest way to move these vaccines ahead into the clinic. So that’s the motivator.” (Dr. Wilson)

„Why do you have to patent? So that companies are interested. And when are firms interested? If there is money to make! [...] I mean as the company Bayer I live from making money and when there is no money made, there is no company Bayer, easy as that” (Prof. Smith)

“So if the HPV vaccine had remained in the academic sector, we wouldn’t have the vaccines today. Nobody would have been able to scrape up the billion necessary for the development” (Prof. Smith)

Financial incentives also played an important role for the participating scientists. These included personal financial profit as well as acquiring funds for their institution or the continuation of their own research. Most participants stated that generating income from commercialization activities has become more important for their PROs and themselves, as public funds for basic research have been reduced. Many participants cited cases where institutions had made a fortune with one key technology and that they believed examples like these were motivating PROs to engage in commercialization and professionalise their TTOs. Some European scientists seemed less enthusiastic, one even saying that licensing revenues do not justify the high cost of filing patents.

“Universities have become much more proactive in working with their scientists to try to patent potentially valuable technologies. […] as funds get harder and harder to come by, government funds for research […] people do look more and more at funding their research by doing things that company may want to fund. And […] the one way to do that is to have some good IP” (Dr. Williams)

“[T]he scientists are also very greedy too, all right? I mean, they really want money, too, [...] there’s really a financial incentive to everybody to go to technology transfer.” (Dr Jones)

“I would estimate that our university makes twenty million dollars a year in patents, royalties. […] It’s not so bad. But some places make, I mean University of Madison Wisconsin had a patent on Vitamin D or something in the early nineties, I mean they made hundreds of millions of dollars. Just off one patent”. (Dr. Jones)

Additionally, one participant stated that his colleague-scientists feel pressured to commercialize their research in order to *“add luster to their resume” (Dr. Williams)*. Overall, they described that patenting and engaging in commercialization had become very common.

“Now people [...] patent everything [...], it’s silly actually. You know, because everybody has become aware of how valuable these patents might be and so, they’ve become hypersensitive to discovery” (Dr. Jones)

### Knowledge exchange and access to medicines

While all interview participants stated that they influence the commercialization of their research results, and that promoting human health was a key motivator for engaging with industry, they did not believe that they could shape their knowledge exchange in a way that would promote global access to the resulting product. Except for one participant who mentioned non-exclusive licenses in the context of public health, public health-sensitive knowledge exchange strategies were not discussed. Instead, a number of interviewees mentioned licensing clauses, such as milestones or non-exclusive licenses they had used to achieve unrelated goals. Nonetheless, as indicated above, participants believed that only exclusive licenses would provide enough security about return on investment to convince pharmaceutical companies to develop a technology. This suggests that they believe the promotion of access to the resulting product would necessarily lead to relevant losses of revenue.

“You could have exclusive versus non [exclusive licenses], and some people, you know, won’t deal with you unless they have an exclusive license because they want to protect their development process, too. So, it is a give and take on both sides and I think that it’s usually, you know, it’s a business decision.” (Dr. Jones)

### Qualitative synthesis: scientist-centric model of public-private knowledge exchange in medical research

**Fig 2:**
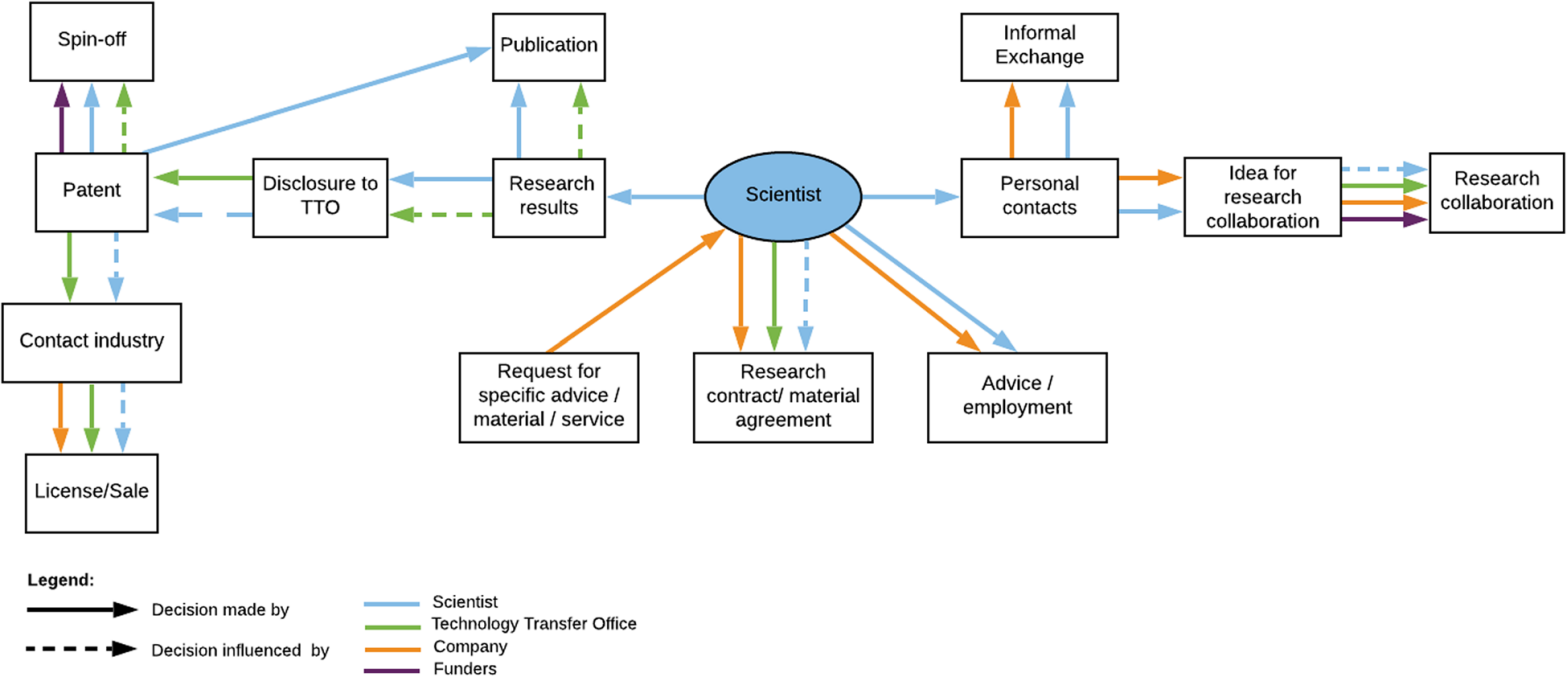
Scientist-centric model of public-private knowledge exchange in medical research

Synthesizing the evidence from both the systematic review and the qualitative interview study, we developed a researcher-centric model for medical knowledge exchange between PROs and industry actors (see Fig. 2).

This model captures four knowledge exchange pathways. First, scientists exchange knowledge through publishing their research results. Secondly, they scan their research results for patentability and disclose what they perceive to be patentable inventions to the PRO TTO. The TTO usually follows through on filing a patent, and often follow suggestions of the inventor scientists with regards to potential commercialization partners and strategies. Formal licensing or sale agreements are negotiated between the TTO and the industry partner, with varying input from the inventors. Instead of licensing, the scientists may initiate spin-off companies, which are either established through the TTO or with the support of other funders. Thirdly, companies seek advice or specific services from PRO scientists, who they often contact directly. Formal agreements for services, material or advice are negotiated between the TTO and the company, but the scientists are often consulted. Foregoing the TTO; scientists may accept paid positions at firms to provide advice and knowledge on a more permanent basis. Finally, scientists exchange knowledge through personal contacts, which may lead to ideas for joint research. After initial discussions, and potential joint publications, scientists bring in the PRO TTO to formalize the collaboration, sometimes even providing draft terms. The agreement is then negotiated between the PRO TTOs and the company, usually including regulations regarding ownership of IP.

## Discussion

### Knowledge exchange in medical research

The results of the systematic review, as well as the interview study, show that the public-private exchange of medical knowledge occurs through four pathway complexes: (1) the publication of research results in journals or at conferences; (2) the transfer of research results through patents and subsequent license, sale, or spin-off companies, (3) problem-specific formal exchange of advice, services, or material; or positions within the company; and (4) exchanges based on informal contacts which may give rise to formalized research collaborations. The scientists themselves were found to be key actors particularly in the early stages of these exchange pathways: Scientists decide whether to publish or patent research results, they are consulted in commercialization efforts by their institutions’ TTOs, they usually initiate the establishment of spin-off companies, and informal contacts established by them are crucial in the development of joint research endeavours. According to our results, their influence lessens over the course of the exchange pathway, although there is evidence that particularly senior scientists are engaged and consulted by the drug developer all the way through to market entry and beyond.

The scientists engaging in the exchange of knowledge in medical research appeared to be highly visible, well connected, male researchers. The motivations to engage with private partners prominently include improving human health through developing and making available new medicines. Financial interests also appeared to be relevant factors, particularly the generation of licensing revenues for academic institutions, although 84% of U.S. university TTOs, in fact, operate at a loss [78]. Similarly, in 2010 TTOs in Germany were supported by the state with a total of €9,6 million, while making only €4,9 million in revenues [79].

The interview results indicate that the knowledge exchange process is driven by individual scientists who see an application for the knowledge they created and initiate knowledge exchange processes with industry. In this, the scienstists approach and connect their institution and potential private partners. Within the Triple Helix Model, such individuals are called “entrepreneurial scientists” [42], researchers who generate scientific knowledge and simultaneously examine it for commercial opportunities. In the qualitative study, we found attitudes and strategies that most closely resembled the “New School” professor described by Owen-Smith and Powell (2001), engaging with industry to promote the practical application of innovation and ultimately public health [67]. Limitations of industry engagement included a focus on applied research as well as conflict of interest. Reflecting these issues, a 2010 study in France found that 33.3% of academics in pharmaceutical research confirmed that patenting opportunities influence their research agenda [80].

### Knowledge exchange and access to medicines

According to the results of the systematic review and interview study, medical scientists were sceptical regarding their ability to engage in public health-sensitive knowledge exchange and unaware of most of the available strategies. In the interview study, this scepticism seemed to stem from four core beliefs: (1) bringing a medical product to market requires “a billion” in investments, (2) promoting affordability necessarily entails significant loss of revenue, (3) in public-private knowledge exchange negotiations, universities have a weak negotiation position, and (4) the development of new medical products is only possible through the involvement of industry actors.

First, the costs associated with developing a drug remain intransparent. The most recent estimate of USD 2,6 billion per approved drug is mostly based on self-reported data provided by pharmaceutical companies [81]. This and most other figures are impossible to verify and have been heavily contested [82–84]. The not-for-profit drug development initiative DNDi has instead suggested that research and development costs range from USD 100–150 million per approved drug, accounting for attrition and risk of failure [84]. These costs cover the entire process from exploratory early discovery - the riskiest of all research stages- to the market. However, at the point where universities begin engaging with industry partners regarding the development of a medical product, they have often progressed beyond the risky, early research stages. In the HPV vaccine case, for example, vaccine candidates had already been developed, their efficacy in animal models had been tested, and production methods had been explored. In cases like these a substantial part of the costs of development have thus already been shouldered by the PROs and ultimately, the public.

Second, the risk of reduced return on investment placed on industry partners through the implementation of public health-sensitive knowledge exchange strategies may be lower than medical scientists appear to believe. According to the WHO, only 18% of the global vaccine market is captured by developing countries [85]. Pushing for affordability of HPV vaccines in LMICs would arguably have limited impact on revenues, and there is no evidence that the differential pricing applied under the Pan American Health Organization’s revolving fund and GAVI has led to re-imports or otherwise affected revenues of manufacturers [86].

Third, pharmaceutical companies increasingly seek and are dependent on interesting technologies emerging from public research institutions to supplement their own pipeline. Many are in fact reducing their in-house early-stage research activities, sometimes with the explicit aim of out-sourcing basic research to public institutions [87–89]. This leads to an increasing dependency of pharmaceutical companies on in-licensing scientific knowledge from public institutions for the development of medicines. As scientists are generally responsible for initiating and designing such agreements, they might thus be in a better position to negotiate alternative transfer strategies than they appear to be aware of.

Finally, there may be alternatives to engaging with private companies in the development of new medicines. In the current drug development system, biomedical and pharmaceutical firms are indeed the only entities developing and producing medical products on a large scale. However, these endeavours are already heavily supported by publicly funded knowledge as well as direct state funds [90, 91]. A not-for-profit drug development program with a scope beyond neglected tropical diseases, such as a global funding mechanism for global health research and development as proposed by the WHO, would offer scientists a chance to turn their research into products without the caveats of industry entanglement and promote access to the resulting medicines. Such measures may enable scientists to support the realization of universal health coverage and access to affordable medicines, key components of the Sustainable Development Goal on Health [22]. It would also help abate some of the side effects of the current drug development system, such as biases in clinical trials, lack of research into new antimicrobials and other unprofitable health issues, as well as increasing financial pressure on social health insurances [92–96]. In addition, the intellectual property regime has so far failed to deliver on its promise of fostering innovation [97, 98].

However, until such changes are made, some public health-sensitive knowledge management strategies that promote access to medicines are already available. In other medical fields, universities are increasingly exploring the implementation of public health-sensitive licensing practices [99, 100] and a set of global health sample clauses extracted from successful agreements can be found on the website of the Association of University Technology Managers [101]. Our results indicate a number of ways in which the implementation of public health-sensitive knowledge exchange strategies could be promoted. First, the scepticism regarding the implementation of public health-sensitive transfer practices among PRO scientists appeared to be based on an incomplete, industry-friendly understanding of the commercialization process. This suggests that education and training on drug development and transfer strategies may enable scientists to be more self-determined and informed when engaging with biomedical and pharmaceutical enterprises. Medical curricula, in particular, should include unbiased education on drug development. Second, institutional policies, or national legislation, should mandate public health-sensitive knowledge exchange in publicly funded research. This study also suggests additional avenues for promoting access to medicines that may be worth exploring. For example, informal exchanges, mature spin-offs looking to sell to a larger firm, or paid positions on advisory boards may be leveraged to promote access to medicines. This could possibly be achieved through institutional or regional standards of conduct for spin-offs or informal communications. Moreover, legal capacity building at the level of PRO TTOs could further strengthen the position and self-determination of PROs and individual scientists when engaging with private partners, whose legal presence appears to be perceived as overwhelming. Lastly, projects such as the University Report Card that monitor universities’ global health practices and evaluate stewardship of IP, could promote transparency and commitment to these issues [102].

### Strengths and limitations

This study represents the first qualitative research on medical technology transfer with a specific focus on access to medicines and vaccines. It draws on a systematic literature review as well as extensive, in-depth interviews that generated detailed data on the commercialization process and key results were consistent across interviews and studies. The study was explorative in nature and followed a case study approach, thus limiting the generalizability of data across other fields of medical research. There is also a risk of social desirability bias, which we aimed to minimize in the conduct of the interviews. The interviews were conducted in German and English, data thus had to be analysed across different languages and quotes had to be translated, possibly affecting the research results. In addition, most interview participants were male; the female perspective on the issues under study, therefore, remains underrepresented. We also chose to focus on the role of the inventor scientist and used a case study of successful commercialization. Building on our research, future studies should include TTO professionals and private actors; and include scientists with less experience in private engagement to triangulate different perspectives. Limitations of the systematic review include that the screening process could not be conducted in duplicate due to resource constraints. However, the screening strategy was piloted on a large portion of the studies in duplicate and all uncertainties regarding the decisions on the remaining studies were discussed within the research team to ensure the quality and rigor of this review.

## Conclusion

From the systematic review and the interview study, we identified publications, formal transfer of patented knowledge, problem-specific advice or service agreements as well as informal exchanges and collaborative research as the main avenues of public-private knowledge exchange in medical research. The inventor scientists played a key role in determining which pathway was adopted and how it was framed, tailoring these processes to achieve the fastest possible translation of their research into marketable products to benefit human health. The involvement of pharmaceutical companies was perceived to be a prerequisite for the successful development of such products and had to be incentivized through lucrative knowledge exchange strategies, such as exclusive licenses. In the interview study, we found scepticism towards the implementation of public health-sensitive knowledge exchange strategies, as these were believed to deter corporate interest in the development of new medicines and thus risk the translation of the scientists’ research.

Discussing these findings within the context of the financial gains and risks as well as the roles currently played by public and private entities in drug development, we find that the views among medical scientists at PROs appear to be biased towards an industry-friendly perspective of medical research and development. We thus suggest that until a delinked model of medical research alleviates the conflict between public and private interests altogether, education of medical students and researchers on the processes, power relations, and financial realities of knowledge exchange and drug development, as well as improved institutional and legal backing, could be key in improving the success of public health-sensitive knowledge exchange strategies.

## Supplementary information

**Additional file 1.**

**Additional file 2.**

**Additional file 3.**

## Data Availability

The datasets generated and/or analysed during the current study are not publicly available due to them containing information that could compromise participant privacy but are available from the corresponding author on reasonable request.

## Abbreviations

DNDi: Drugs for neglected disease initiative
GAVI: Gavi, the Vaccine Alliance
IP: Intellectual Property
LMIC: Low and Middle Income Countries
PRO: Public Research Organization
TTO: Technology Transfer Office
UAEM: Universities Allied for Essential Medicines
WHO: World Health Organization

## References

[1] Stevens AJ, Jensen JJ, Wyller K, Kilgore PC, Chatterjee S, Rohrbaugh ML (2011). The role of public-sector research in the discovery of drugs and vaccines. New Engl J Med.

[2] Chatterjee SK, Rohrbaugh ML (2014). NIH inventions translate into drugs and biologics with high public health impact. Nat Biotechnol.

[3] Weyerstahl T, Stauber M. Gynäkologie und Geburtshilfe. 3rd ed. Stuttgart: Georg Thieme Verlag; 2013.

[4] Richens J, Mabey DCW. Sexually transmitted infections. In: Cook GC, Zumla AI, editors. Manson's tropical diseases. 22nd ed. Philadelphia: Saunders Elsevier; 2009. p. 403–35.

[5] Bray F, Ferlay J, Soerjomataram I, Siegel RL, Torre LA, Jemal A (2018). Global cancer statistics 2018: GLOBOCAN estimates of incidence and mortality worldwide for 36 cancers in 185 countries. CA-Cancer J Clin.

[6] Oparka R, Herrington CS. Human papillomavirus infection and its association with Neoplasia: from molecular biology to prevention and treatment. In: Gaston K, editor. Small DNA Tumor Viruses. Poole: Caister Academic Press; 2012. p. 1–19.

[7] Sankaranarayanan R, Anorlu R, Sangwa-Lugoma G, Denny LA (2013). Infrastructure requirements for human papillomavirus vaccination and cervical cancer screening in sub-Saharan Africa. Vaccine..

[8] McIntyre P. Finding the viral link: the story of Harald zur Hausen. Cancerworld. 2005; http://cancerworld.net/wp-content/uploads/2017/09/6737_cw7_32_37_Masterpiece-2.pdf. .

[9] Grimes J (2006). HPV vaccine development: A case study of prevention and politics. Biochem Mol Biol Edu.

[10] Ghim S-J, Jenson AB, Schlegel R (1992). HPV L1 protein expressed in cos cells displays conformational epitopes found on intact virions. J Virol.

[11] Kirnbauer R, Booy F, Cheng N, Lowy DR, Schiller JT (1992). Papillomavirus L1 major capsid protein self-assembles into virus-like particles that are highly immunogenic. Proc Natl Acad Sci U S A.

[12] Rose RC, White WI, Li M, Suzich JA, Lane C, Garcea RL (1998). Human papillomavirus type 11 recombinant capsomeres induce virus-neutralizing antibodies. J Virol.

[13] University of Rochester. http://www.urmc.rochester.edu/cancer-center/researchers/research-accomplishments/cervical-cancer-vaccine.aspx Accessed 2 Dec 2018.

[14] Suzich JA, Ghim SJ, Palmer-Hill FJ, White WI, Tamura JK, Bell JA (1995). Systemic immunization with papillomavirus L1 protein completely prevents the development of viral mucosal papillomas. Proc Natl Acad Sci U S A.

[15] Schiller JT, Lowy DR (1996). Papillomavirus-like particles and HPV vaccine development. Semin Cancer Biol.

[16] Crager Sara E., Guillen Ethan, Price Matt (2009). University Contributions to the HPV Vaccine and Implications for Access to Vaccines in Developing Countries: Addressing Materials and Know-How in University Technology Transfer Policy. American Journal of Law & Medicine.

[17] Padmanabhan S, Amin T, Sampat B, Cook-Deegan R, Chandrasekharan S (2010). Intellectual property, technology transfer and manufacture of low-cost HPV vaccines in India. Nat Biotech.

[18] Clark A. Biomedical innovation and the politics of scientific knowledge: a case study of Gardasil. University of Maryland. 2008.

[19] World Health Organization. 21th WHO Model List of Essential Medicines. 2019. https://apps.who.int/iris/bitstream/handle/10665/325771/WHO-MVP-EMP-IAU-2019.06-eng.pdf?ua=1. Accessed 10 Sept 2019.

[20] International Covenant on Economic, Social and Cultural Rights, A/RES/2200A [XXI]. United Nations General Assembly; 1966. https://www.un.org/en/development/desa/population/migration/generalassembly/docs/globalcompact/A_RES_2200A(XXI)_civil.pdf. Accessed 9 Mar 2019.

[21] United Nations Secretary-General's High-Level Panel on Access to Medicines Report. United Nations Secretary-General's High-Level Panel on Access to Medicines. 2016. http://www.unsgaccessmeds.org/final-report. Accessed 20 Apr 2018.

[22] Progress towards the Sustainable Development Goals (E/2016/75). United Nations; 2016. https://www.un.org/ga/search/view_doc.asp?symbol=E/2016/75&Lang=E. Accessed 10 May 2019.

[23] Wirtz Veronika J, Hogerzeil Hans V, Gray Andrew L, Bigdeli Maryam, de Joncheere Cornelis P, Ewen Margaret A, Gyansa-Lutterodt Martha, Jing Sun, Luiza Vera L, Mbindyo Regina M, Möller Helene, Moucheraud Corrina, Pécoul Bernard, Rägo Lembit, Rashidian Arash, Ross-Degnan Dennis, Stephens Peter N, Teerawattananon Yot, 't Hoen Ellen F M, Wagner Anita K, Yadav Prashant, Reich Michael R (2017). Essential medicines for universal health coverage. The Lancet.

[24] de Martel C, Ferlay J, Franceschi S, Vignat J, Bray F, Forman D (2012). Global burden of cancers attributable to infections in 2008: a review and synthetic analysis. Lancet Oncol.

[25] Forman D, de Martel C, Lacey CJ, Soerjomataram I, Lortet-Tieulent J, Bruni L (2012). Global Burden of Human Papillomavirus and Related Diseases. Vaccine.

[26] Bruni L, Diaz M, Barrionuevo-Rosas L, Herrero R, Bray F, Bosch FX (2016). Global estimates of human papillomavirus vaccination coverage by region and income level: a pooled analysis. Lancet Glob Health.

[27] Gallagher KE, LaMontagne DS, Watson-Jones D (2018). Status of HPV vaccine introduction and barriers to country uptake. Vaccine.

[28] Hutubessy R, Levin A, Wang S, Morgan W, Ally M, John T (2012). A case study using the United Republic of Tanzania: costing nationwide HPV vaccine delivery using the WHO cervical Cancer prevention and control costing tool. BMC Med.

[29] Kaddar M, Schmitt S, Makinen M, Milstien J (2013). Global support for new vaccine implementation in middle-income countries. Vaccine.

[30] Clendinen C, Zhang Y, Warburton RN, Light DW (2016). Manufacturing costs of HPV vaccines for developing countries. Vaccine..

[31] AccessCampaign MSF (2017). A fair shot for vaccine affordability: understanding and addressing the effects f patents on access to newer vaccines.

[32] Herlihy N, Hutubessy R, Jit M (2016). Current global pricing for human papillomavirus vaccines brings the greatest economic benefits to rich countries. Health Aff.

[33] GlaxoSmithKline plc. Annu Rep. 2017:2018 https://www.gsk.com/media/4751/annual-report.pdf.

[34] Access to medicines and vaccines: Report by the Director-General [A72/17]. World Health Organization; 2019. http://apps.who.int/gb/ebwha/pdf_files/WHA72/A72_17-en.pdf?ua=1. Accessed 20 May 2019.

[35] Godt C. Equitable Licensing & Global Access: Lizenzpolitik & Vertragsbausteine. 2nd ed. Bielefeld: BUKO Pharma-Kampagne; 2017.

[36] Chen CE, Gilliland CT, Purcell J, Kishore SP (2010). The silent epidemic of Exclusive University licensing policies on compounds for neglected diseases and beyond. PLoS Neglect Trop D.

[37] Guebert JM, Bubela T (2014). Implementing Socially Responsible Licensing for Global Health: Beyond Neglected Diseases. Sci Transl Med.

[38] Nelsen L (2013). The role of university technology transfer operations in assuring access to medicines and vaccines in developing countries. Yale J Health Policy Law Ethics.

[39] Yale University Center for Interdisciplinary Research on AIDS. Access to essential medicines and university research: building best practices. Yale University; 2003. http://cira.med.yale.edu/. Accessed 21 June 2017.

[40] BUKO Pharmakampagne (2018). Pharmabrief Spezial - Leitfaden für sozial-verträgliche Verwertung - Forschungsergebnisse für möglichst viele Menschen nutzbar machen.

[41] Etzkowitz H, Leydesdorff L (2000). The dynamics of innovation: from National Systems and “mode 2” to a triple Helix of university–industry–government relations. Res Policy.

[42] Ranga M, Etzkowitz H (2013). Triple Helix systems: an analytical framework for innovation policy and practice in the knowledge society. Ind High Educ.

[43] Bradley S, Hayter C, Link A. Models and methods of university technology transfer. UNCG economics working paper series 13-10. Greensboro: University of North Carolina; 2013.

[44] Perkman M, Tartari V, McKelvey M, Autio E, Broström A, D’Este P (2013). Academic engagement and commercialization: a review of the literature on university-industry relations. Res Policy.

[45] Etzkowitz H (1998). The norms of entrepreneurial science: cognitive effects of the new university–industry linkages. Res Policy.

[46] Jahn R, Müller O, Bozorgmehr K (2015). Characteristics and determinants of knowledge transfer policies at universities and public institutions in medical research—protocol for a systematic review of the qualitative research literature. Systematic Reviews.

[47] Joanna Briggs Institute. Joanna Briggs Institute Reviewer’s Manual 2014. http://joannabriggs.org/assets/docs/sumari/ReviewersManual-2014.pdf. Accessed 14 Oct 2014.

[48] Black N (1994). Why we need qualitative research. J Epidemiol Commun H.

[49] The Clinical Appraisal Skill Programme. Qualitative Research Checklist. http://www.caspinternational.org/mod_product/uploads/CASP%20Qualitative%20Research%20Checklist%2031.05.13.pdf. Accessed 14 Oct 2014.

[50] Campbell R, Pound P, Morgan M, Daker-White G, Britten N, Pill R (2011). Evaluating meta-ethnography: systematic analysis and synthesis of qualitative research. Health Technol Assess.

[51] Erickson BK, Landers EE, Huh WK (2014). Update on vaccination clinical trials for HPV-related disease. Clin Ther.

[52] Gersch Elizabeth D, Gissmann Lutz, Garcea Robert L (2011). New approaches to prophylactic human papillomavirus vaccines for cervical cancer prevention. Antiviral Therapy.

[53] Kanda T, Kondo K (2009). Development of an HPV vaccine for a broad spectrum of high-risk types. Hum Vaccines.

[54] Kondo K, Ochi H, Matsumoto T, Yoshikawa H, Kanda T (2008). Modification of human papillomavirus-like particle vaccine by insertion of the cross-reactive L2-epitopes. J Med Virol.

[55] Cho H-J, Oh Y-K, Kim YB (2011). Advances in human papilloma virus vaccines: a patent review. Expert Opin Ther Pat.

[56] Moscicki A-B (2008). HPV Vaccines: Today and in the Future. J Adolesc Health.

[57] Tyler M, Tumban E, Chackerian B (2014). Second-generation prophylactic HPV vaccines: successes and challenges. Expert Rev Vaccines.

[58] Rybicki EP (2009). Plant-produced vaccines: promise and reality. Drug Discov Today.

[59] Nieto K, Weghofer M, Sehr P, Ritter M, Sedlmeier S, Karanam B (2012). Development of AAVLP (HPV16/31L2) particles as broadly protective HPV vaccine candidate. PLoS One.

[60] Tumban E, Peabody J, Tyler M, Peabody DS, Chackerian B (2012). VLPs displaying a single L2 epitope induce broadly cross-neutralizing antibodies against human papillomavirus. PLoS One.

[61] Schellenbacher C, Kwak K, Fink D, Shafti-Keramat S, Huber B, Jindra C (2013). Efficacy of RG1-VLP vaccination against infections with genital and cutaneous human papillomaviruses. J Invest Dermatol.

[62] Jagu S, Kwak K, Karanam B, Huh WK, Damotharan V, Chivukula SV (2013). Optimization of Multimeric human papillomavirus L2 vaccines. PLoS One.

[63] Buse K, Mays N, Walt G (2012). Making health policy.

[64] Ritchie J, Spencer L. Qualitative data analysis for applied research. In: Huberman AM, Miles MB, editors. The qualitative researcher's companion. Thousand Oaks: SAGE Publications Inc; 2002. p. 305–29.

[65] Ard CF. The commercialization of clinical genetic technologies: a technology assessment using fluorescence in situ hybridization as the genetic lens [dissertation]. Brandeis University. 2002.

[66] Bacevice PA (2010). Small world, big ideas, and smart companies–a qualitative study of academic spin-off companies and knowledge creation: University of Michigan.

[67] Owen-Smith J, Powell WW. Careers and contradictions: Faculty responses to the transformation of knowledge and its uses in the life sciences. in Steven Avvals, editor. The Transformation of Work (Research in the Sociology of Work, Vol 10). Emerald Group Publishing Limited, 2001. p.109–140..

[68] Villasana M (2011). Fostering university-industry interactions under a triple helix model: the case of Nuevo Leon. Mexico Sci Publ Policy.

[69] Wadmann S (2014). Physician–industry collaboration: conflicts of interest and the imputation of motive. Soc Stud Sci.

[70] Dooley L, Kirk D, Philpott K (2013). Nurturing life-science knowledge discovery: managing multi-organisation networks. Prod Plan Control.

[71] Filieri R, McNally RC, O'Dwyer M, O'Malley L (2014). Structural social capital evolution and knowledge transfer: evidence from an Irish pharmaceutical network. Ind Mark Manag.

[72] Lander B, Atkinson-Grosjean J (2011). Translational science and the hidden research system in universities and academic hospitals: a case study. Soc Sci Med.

[73] Roback K. Medical device innovation: the integrated processes of invention, diffusion and deployment [dissertation]: Linköping University; 2006.

[74] Colaianni A, Cook-Deegan R (2009). Columbia University's Axel patents: technology transfer and implications for the Bayh-dole act. Milbank Q.

[75] Styhre A (2014). Coping with the financiers: attracting venture capital investors and end-users in the biomaterials industry. Technol Anal Strateg.

[76] Shah R, Singer PA, Daar AS (2010). Science-based health innovation in Tanzania: bednets and a base for invention. BMC int health human r.

[77] Rowe S, Alexander N, Kretser A, Steele R, Kretsch M, Applebaum R (2013). Principles for building public-private partnerships to benefit food safety, nutrition, and health research. Nutr Rev.

[78] Valdivia WD. University start-ups: critical for improving technology transfer. Center for Technology Innovation at Brookings Washington, DC: Brookings Institution. 2013.

[79] Cuntz A, Dauchert H, Meurer P, Philipps A, Platz P (2012). Hochschulpatente zehn Jahre nach Abschaffung des Hochschullehrerprivilegs. Studien zum deutschen Innovationssystem Nr 13–2012.

[80] Penin J (2010). On the consequences of Patenting University research: lessons from a survey of French academic inventors. Ind Innov.

[81] DiMasi JA, Grabowski HG, Hansen RW (2016). Innovation in the pharmaceutical industry: new estimates of R&D costs. J Health Econ.

[82] Morgan S, Grootendorst P, Lexchin J, Cunningham C, Greyson D (2011). The cost of drug development: a systematic review. Health policy.

[83] Hirschler B. GlaxoSmithKline boss says new drugs can be cheaper. Reuters. 2013; https://www.reuters.com/article/us-glaxosmithkline-prices/glaxosmithkline-boss-says-new-drugs-can-be-cheaper-idUSBRE92D0RM20130314. .

[84] DNDi.Research & Development for Diseases of the Poor: A 10-Year Analysis of Impact of the DNDi Model [press release]. 2013. https://www.dndi.org/2013/media-centre/press-releases/dndi-rd-model/ Accessed 2 Dec 2018.

[85] World Health Organization. Vaccine Market 2016 http://www.who.int/immunization/programmes_systems/procurement/market/global_demand/en/. Accessed 2 Dec 2018.

[86] Plahte J (2005). Tiered pricing of vaccines: a win-win-win situation, not a subsidy. Lancet Infect Dis.

[87] Rafols I, Hopkins MM, Hoekman J, Siepel J, O'Hare A, Perianes-Rodríguez A (2014). Big Pharma, little science?: a bibliometric perspective on big Pharma's R&D decline. Technol Forecast Soc.

[88] AstraZeneca. AstraZeneca Annual Report 2010. London: AstraZeneca; 2010. https://www.astrazeneca.com/content/dam/az/Investor_Relations/annual-reports-homepage/2010-Annual-Report-English.pdf. .

[89] Moses H, Matheson DM, Cairns-Smith S, George BP, Palisch C (2015). The anatomy of medical research: US and international comparisons. JAMA..

[90] Kneller R (2010). The importance of new companies for drug discovery: origins of a decade of new drugs. Nat Rev Drug Discov.

[91] Sampat BN, Lichtenberg FR (2011). What are the respective roles of the public and private sectors in pharmaceutical innovation?. Health Affair.

[92] Marston HD, Dixon DM, Knisely JM, Palmore TN, Fauci AS (2016). Antimicrobial resistance. JAMA..

[93] Lexchin J (2012). Those who have the gold make the evidence: how the pharmaceutical industry biases the outcomes of clinical trials of medications. Sci Eng Ethics.

[94] Littmann J, Viens AM (2015). The ethical significance of antimicrobial resistance. Public Health Eth.

[95] World Health Organization. Research and Development to meet health needs in developing countries: strengthening global financing and coordination. Report of the consultative expert working group on research and development: financing and coordination. World Health Organization; 2012. https://www.who.int/phi/CEWG_Report_5_April_2012.pdf. Accessed 27 Apr 2019.

[96] European Commission. Report from the Commission to the Council and the European Parliament: Competition Enforcement in the Pharmaceutical Sector, European competition authorities working together for affordable and innovative medicines. Luxembourg; 2019. https://ec.europa.eu/competition/publications/reports/kd0718081enn.pdf Accessed 10 Sept 2019.

[97] Baker D, Jayadev A, Stiglitz J (2017). Innovation, intellectual property, and development: A better set of approaches for the 21st century.

[98] Stiglitz JE, Jayadev A (2010). Medicine for tomorrow: some alternative proposals to promote socially beneficial Research and Development in pharmaceuticals. Journal of Generic Medicines: The Business Journal for the Generic Medicines Sector.

[99] Universities Allied For Essential Medicines (2017). RE:ROUTE: A map of the alternative biomedical R&D landscape.

[100] Chokshi DA (2006). Improving access to medicines in poor countries: the role of universities. PLoS med.

[101] The AUTM Global Health Toolkit: AUTM. https://autm.net/surveys-and-tools/tools/global-health-toolkit .Accessed 2 Dec 2018.

[102] Universities Allied For Essential Medicines. University Report Card: Global Equity and Biomedical Research. http://globalhealthgrades.org/. Accessed 2 Dec 2018.

