# Supplementary material for: Public-private knowledge transfer and access to medicines: a systematic review and qualitative study of perceptions and roles of scientists involved in HPV vaccine research
Source: Global Health. 2020 Mar 5;16:22. doi: 10.1186/s12992-020-00552-9 (PMC7059709; doi:10.1186/s12992-020-00552-9)
Supplement: Supplementary file 1 — Additional file 1. [file 12992_2020_552_MOESM1_ESM.pdf]

Interview Guide (first)

1. Sociodemographic Data

**Sex**	
**Age**	
**Researching HPV for how many years?**	
**Position**	

2. Topics

Context – factors influencing decision:

- Research
  - Characteristics of research topic, results, research group
  - Characteristics of research process – collaboration with academic and non-academic partners
- University
  - Faculty attitude and culture
    - Procedure - technology transfer / Reward system
    - Information - about licensing options, technology transfer
  - TTO
    - Attitude, ease of working with TTO,…
- Law - Legal background, ownership of results, federal policies encouraging transfer
- Economic - Funding of research / faculty – private / public / royalties / licensing fees
- Local - If Industry/institutes nearby – collaboration?
- Personal
  - Incentives - technology transfer, patents, access to innovation…
  - Opinion - interest in commercialization, value of patents / publications…
  - Knowledge - about technology transfer process / licensing options…

Content – Technology transfer:

- Formal - patent / copyright, licences, research agreements/collaborations
- Informal - meetings, joint publications, conferences, etc. (how many?)
- Relative importance of both, relationship between the two (eg. temporal)

Actors – Who decides?

- Legally, factually, personal role

Process – How do they decide?

- Consensus-building at the University
- Negotiations with Industry

Interview Guide (final)

1. Sociodemographic Data

**Sex**	
**Age**	
**Researching HPV for how many years?**	
**Position**	

2. Topics

1. Content – How do you transfer knowledge?
- a. Formally and informally
- b. Relative importance of transfer pathways

2. Actors – Who decides how to transfer research results?
- a. Legally, factually, personal role
- b. Negotiation position – representation, importance, power of each actor?
- c. Characteristics of actors
- d. What is important for you / your institution when you transfer knowledge?

3. Process – What is the decision-making process at the institution?
- a. Building a consensus at the institution
- b. Negotiations with industry
- c. Research collaborations

4. Context – What external factors influence this decision?
- a. Research – Type of research, type of result
- b. Juridicial
- c. Economic
- d. Ethical implications

5. If appropriate:
Now that we've covered the general process, I'd like to ask you a few additional questions:
- a. Informal transfer – content, actors, process
- b. Change – how has the knowledge transfer changed?
- c. Impact - What impact does the commercialisation of research have on public research?
- d. Access to medicines – Possible influence of knowledge transfer? What do you think about socially responsible licensing?
