# Supplementary material for: Public-private knowledge transfer and access to medicines: a systematic review and qualitative study of perceptions and roles of scientists involved in HPV vaccine research
Source: Global Health. 2020 Mar 5;16:22. doi: 10.1186/s12992-020-00552-9 (PMC7059709; doi:10.1186/s12992-020-00552-9)
Supplement: Supplementary file 2 — Additional file 2. [file 12992_2020_552_MOESM2_ESM.pdf]

Coding Tree

**CONTEXT**			
**Scientific**			
	Public vs private research		
	Characteristics of research finding		
		Specific regulatory issues	
		Potential profit	
		Applicability	
		Patentability	
		scientific	
		potential public health benefit	
		Complexity	
	Characteristics of research area		
		Relevance for developing countries	
		Competitiveness	
		basic vs applied	
	Research community		
**R&D funding system**			
**Public Opinion**			
**Societal**			
	Prices		
	Other		
**Legal regulations**			
**ACTORS**			
**Scientist**			
	Personal characteristics		
		career specifics	
		Resources	
		Motivation	
			Can't develop alone
			Protect your intellectual property
			Get resources for further research
			Improve human health
			Interest, fun
			Financial incentive
			other
		Age	
		Personality	
	Negotiating position		
		Post-license	
		spin-off	
		Patenting decision	
		Finding company	
		Negotiation w/ company	
		other	
**Institution**			
	Institutional characteristics		
		tech transfer policies	
		reputation	
		Size	
		type - govt, uni, private, public	
		Resources	
			Patent revenues important source of income
			Knowledge
			Financial resources
			Legal resources
	Negotiating position		
		Equality	
		Legal resources	
**Companies**			
	Characteristics		
		Location	
		Resources	

			human resources
			financial
		Motivation	
			profit
		Scientific work	
			industrial research: highly specialised on very few processes
		Organisation	
			Ease of communication/ finding the right person to talk to
			Changing management of the company
		nationality	
		size	
	negotiating position		
**Funders**			
**<u>CONTENT</u>**			
**Publications**			
	encouraged not to publish if wish to patent		
	publish includes poster presentations, talks		
**Joining an existing firm**			
**Advice /Service for companies**			
	Service agreement		
	Providing test/model/compound to a company		
	Confidentiality agreements		
	Expert reports		
**Research agreements**			
**Patents**			
	types of patents		
	Patenting cost		
	Patent revenues		
	Ownership of IP		
**Licensing agreements**			
	down-payment, share of profit		
	Milestones in licensing agreements		
	Non-exclusive licenses		
	License - field of use		
	Exclusive license		
**Spin-off**			
	aim: develop IP and then sell		
	spin-off very common, epidemic		
**Other**			
	Differential pricing for access		
	WHO advisor		
	Company scientist visits lab		
**Research grants**			
**Informal transfer**			
	talks at conferences/meetings		
	Informal advice to companies directly		
**Collaborative research in general**			
**Relative importance of TT pathway**			
	Money for patent determines the worth of research		
	Publication more important than patent		
	Black mark if you miss opportunity to patent		
	Peer review process makes article more valuable than patent		
	Peer reviewed paper more important that patents for career		
**<u>PROCESS</u>**			
**Patents/Licenses**			
	Early research stages		
		In early stages: naive, open exchange	
	Discussing results with research group		
	Disclosing an invention		
	Patenting		
		Decision-making	
			Interaction TTO - scientist
			Interaction TTO - institution
		Patenting process	

			Institution applies for patent
			If institution doesn't patent --> researcher can file
			If patent not followed up -->public domain
	Marketing the patent, finding a company		
		Marketing the patent	
		Financial pressure to find a buyer quickly or give up patent	
		Establishing contact with a company	
	Negotiating an agreement with industry		
		Using TTO as hard bargainer	
		Negotiating a price	
		Other	
	Founding a spin-off		
		funding	
		dog and pony show	
		influence of inventor	
**Research agreements**			
	Establishing contact with companies		
		not organized, word of mouth	
		based on publication	
		other	
	Writing contract		
		confidentiality agreement	
		other	
	Applying for industry funding		
**Collaboration in general**			
**<u>Evaluation</u>**			
**Neutral**			
	Evaluation is difficult - time will tell		
	No negative personal consequences of patenting		
**Positive**			
	Working with companies offers new perspectives		
	Patent revenues		
	IP protection necessary to incentivize companies to develop		
	No development possible without companies, need to engage		
	Companies pre-select promising innnovations		
	Collaborations are important and can be fruitful		
	commercialization without partner requires boring work		
	patent revenues pay for research		
	Spin-offs are a good way of developing an invention		
**Negative**			
	The more money involved the more problematic		
	Conflicts in collaborations with companies		
	Contract research limits freedom to do what's interesting		
	Different interests - comp and scientist		
	Comp license IP to keep it off the market		
	Unreasonable patenting leads to problems		
	Patents are expensive und often unexploited - not worth it		
	Patenting delays publication		
**Evaluation of specific transfers**			
	Goal to achieve access in developing countries not yet realized		
	Pleased with outcome of HPV vaccine transfer process		
**<u>Change</u>**			
**What has changed?**			
	Patenting more common, increased awareness		
	Less tech transfer, more collaborative research		
	Unis/inst's have more expertise, experience, policies, structur		
	Opinion on commercialization - used to be seen as negative		
	No change		
	Unis and comp. less willing to take risks, translation harder		
	Old days - scientists always protected IP - eg publication		
**Why has it changed?**			

	TTOs, scientists now know patents are valuable
	negative experience with unprot. IP -> more patenting
	TTOs more sophisticated
**Assessment of change**	
	neutral
	positive
	negative
**Impact**	
	Time
	Conflict of interest
	More thinking about implementation issues, cost-effectiveness
	chicken and egg - more translational research <> more transfer
	No impact
	Other
