# Supplementary material for: Public-private knowledge transfer and access to medicines: a systematic review and qualitative study of perceptions and roles of scientists involved in HPV vaccine research
Source: Global Health. 2020 Mar 5;16:22. doi: 10.1186/s12992-020-00552-9 (PMC7059709; doi:10.1186/s12992-020-00552-9)
Supplement: Supplementary file 3 — Additional file 3. [file 12992_2020_552_MOESM3_ESM.pdf]

Systematic Review – PRISMA Flow Chart and description of included studies

(1) PRISMA Flow Chart

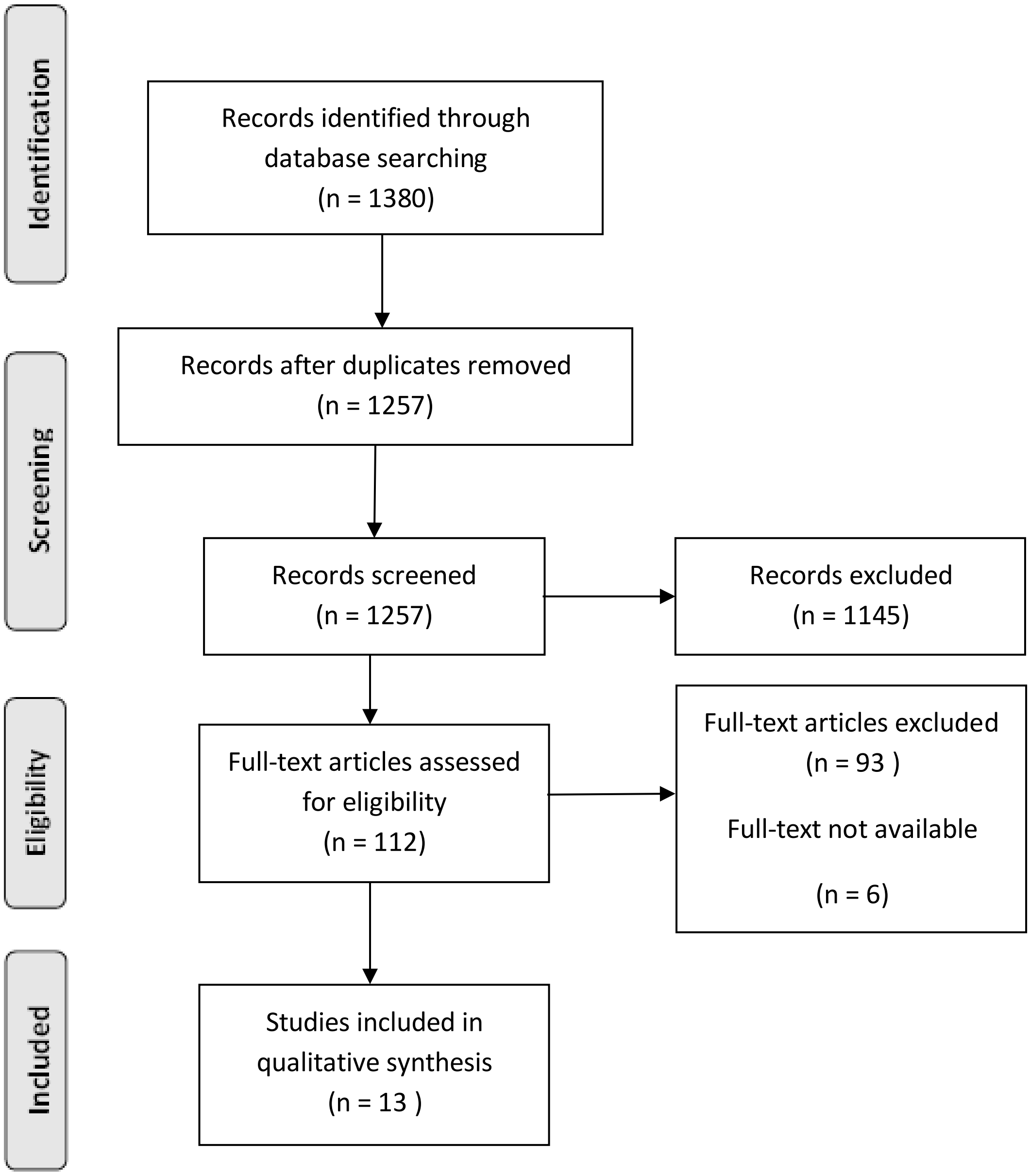

(2) Description of studies included in the systematic review

Authors	Year	Title	Objective is to study…	Methods, (CASP score)	Study population	Location
Ard	2003	**The commercialization of clinical genetic technologies: A technology assessment using fluorescence in situ hybridization as the genetic lens (dissertation)**	...the roles of academia, government, and the U.S. biotechnology industry, in the research, development, and diffusion of genetics research	Case study, semi-structured interviews, (9)	1 academic scientist, 2 genetics professors, 1 clinical genetics professional, 2 directors of a clinical genetics center, 1 senior level university administrator, 2 senior biotech executives	USA
Bacevice	2010	**Small World, Big Ideas, and Smart Companies - A Qualitative Study of Academic Spin-off Companies and Knowledge Creation (dissertation)**	…how spin-offs create knowledge, the ways in which knowledge flows both within and outside of the company	Case study, semi-structured inter-views, 2008-2009 (9)	18 individuals affiliated with 6 academic spin-offs in the life sciences or medicine	Australia
Filieri et al	2014	**Structural social capital evolution and knowledge transfer: Evidence from an Irish pharmaceutical network**	…how the structure of industry–university networks evolves and influences knowledge transfer	Single-site case study, interviews (8)	7 academics and 11 industry managers directly involved in the activities of the network	Ireland
Owen-Smith & Powell	2004	**Careers and contradictions: Faculty responses to the transformation of knowledge and its uses in the life sciences**	…how faculty who are differently positioned with regard to research commercialization are developing varied interpretations of what careers and research programs look like	>70 semi-structured and 15 informal interviews, fieldwork (8)	Prestigious and prolific academic scientists and research administrators in the life sciences	USA
Styhre	2014	**Coping with the financiers: attracting venture capital investors and end-users in the biomaterials industry**	...the challenges in establishing university spin-outs	Case study, interviews (8)	4 male and 4 female researchers and administrative staff at two university spin-outs involved in orthopedic device development	Sweden
Rowe et al	2013	**Principles for building public-private partnerships to benefit food safety, nutrition, and health research**	...principles for effective public-private partnerships (PPPs) in scientific research	Semi-structured interviews (7)	Senior individuals from 27 organisations currently engaged in public-private partnerships, including academic scientists	Global

Schah, Singer & Daar	2010	**Science-based health innovation in Tanzania: bednets and a base for invention**	…Tanzania's science-based health innovation system	Case study, semi-structured interviews, 2007 (7)	16 government officials, 22 researchers, 6 entrepreneurs, 2 NGO representatives	Tanzania
Wadmann	2014	**Physician-industry collaboration: conflicts of interest and the imputation of motive**	…how policies regarding innovation and conflict of interest interact with everyday practice in clinical hypertension research in Denmark	Ethnographic fieldwork, 22 semi-structured interviews (7)	10 physician investigators, 8 research nurses, 3 pharmaceutical company executives, 1 chief economist from the Danish Pharmaceutical Industry association	Denmark
Dooley, Kirk & Philpott	2015	**Nurturing life-science knowledge discovery: managing multi-organisation networks**	… how knowledge networks linking public research centres and pharmaceutical organisations can be managed to deliver benefits	Case study, semi-structured inter-views, 2004-2008 (6)	19 academics, 5 industry members of a management team of a structured, long-term collaboration in medical research	Not specified
Colaianni & Cook-Deegan	2009	**Columbia University's Axel Patents: Technology Transfer and Implications for the Bayh-Dole Act**	…the effect of the Bayh-Dole Act on incentives for federal grantees to pursue royalty revenues from patented research	Interviews, 2005 (5)	2 inventors named on Columbia's Axel patents	New York City, USA
Lander & Atkinson-Grosjean	2011	**Translational science and the hidden research system in universities and academic hospitals: A case study**	…the 'hidden research system' that connects hospitals and universities with the clinical and scientific actors who make the linkages possible and individual interactions and dynamics involved in immune system research	Case study, structured & semi-structured interviews, participant-observer, 2007-2009 (5)	16 clinician-scientists and their teams and 4 collaborating university scientists in a network working on IRAK-4 deficiency research	Not specified
Roback, Hass & Persson	2001	**Transfer of health care technology in university-industry research collaboration environment**	…the research process in the field of biomedical engineering and to identify determinants of technology transfer	Semi-structured interviews (5)	Senior researchers at 11 projects between academia and industry in health research at the Competence Center NIMED at Linköping university, Sweden	Linköping, Sweden
Villasana	2011	**Fostering university-industry interactions under a triple helix model: the case of Nuevo Leon, Mexico**	…how academic researchers develop interactions with industry	Semi-structured interviews (5)	16 faculty performing research in biotechnology at two universities (UANL and ITESM) in Nuevo Leon	Nuevo Leon, Mexico
